# Knowledge and practices of general practitioners at district hospitals towards cervical cancer prevention in Burundi, 2015: a cross-sectional study

**DOI:** 10.1186/s12992-018-0321-5

**Published:** 2018-01-16

**Authors:** Zacharie Ndizeye, Davy Vanden Broeck, Heleen Vermandere, John Paul Bogers, Jean-Pierre Van Geertruyden

**Affiliations:** 1 Faculty of Medicine, Community Medicine Department, University of Burundi, Bujumbura, Burundi; 2 Faculty of Medicine and health sciences, Global Health Institute, University of Antwerp, Antwerp, Belgium; 3 International Centre for Reproductive Health, Ghent University, Ghent, Belgium; 4 Laboratory of Molecular Pathology, AML, Antwerp, Belgium; 5 AMBIOR, Laboratory for Cell Biology & Histology, University of Antwerp, Antwerp, Belgium

**Keywords:** Knowledge, Practices, General practitioners, Cervical cancer prevention

## Abstract

**Background:**

Well-organized screening and treatment programmes are effective to prevent Invasive Cervical Cancer (ICC) in LMICs. To achieve this, the World Health Organization (WHO) recommends the involvement of existing health personnel in casu doctors, nurses, midwives in ICC prevention. A necessary precondition is that health personnel have appropriate knowledge about ICC. Therefore, to inform policy makers and training institutions in Burundi, we documented the knowledge and practices of general practitioners (GPs) at district hospital level towards ICC control.

**Methods:**

A descriptive cross-sectional survey was conducted from February to April, 2015 among all GPs working in government district hospitals. A structured questionnaire and a scoring system were used to assess knowledge and practices of GPs.

**Results:**

The participation rate was 58.2%. Majority of GPs (76.3%) had appropriate knowledge (score > 70%) on cervical cancer disease; but some risk factors were less well known as smoking and the 2 most important oncogenic HPV. Only 8.4% of the participants had appropriate knowledge on ICC prevention: 55% of the participants were aware that HPV vaccination exists and 48.1% knew cryotherapy as a treatment method for CIN. Further, 15.3% was aware of VIA as a screening method. The majority of the participants (87%) never or rarely propose screening tests to their clients. Only 2 participants (1.5%) have already performed VIA/VILI. Wrong thoughts were also reported: 39.7% thought that CIN could be treated with radiotherapy; 3.1% thought that X-ray is a screening method.

**Conclusion:**

In this comprehensive assessment, we observed that Burundian GPs have a very low knowledge level about ICC prevention, screening and treatment. Suboptimal practices and wrong thoughts related to ICC screening and treatments have also been documented. We therefore recommend an adequate pre- and in-service training of GPs and most probably nurses on ICC control before setting up any public health intervention on ICC control.

## Background

Worldwide, invasive cervical cancer (ICC) is the fourth most common female cancer after breast cancer, colorectal cancer and lung cancer [1]. In 2012, the number of new cases was estimated at 528000, corresponding to an age-standardised incidence rate of 14 per 100,000 women. The number of deaths attributed to ICC was estimated at 266000, corresponding to an age-standardised mortality rate of 6.8/100000 women [1]. Huge disparities in ICC control exist between High and Low & Middle Income Countries (LMICs), mainly due to the limited human capacity and infrastructure and other competing priorities in LMICs [2–4]. Around 85% of new ICC cases and 88% of ICC related deaths occurred in LMICs mainly in sub-Saharan Africa and South-East Asia [1, 2, 5]. In sub-Saharan Africa, in particular in Eastern Africa, ICC is the most common female cancer with an age-standardised incidence and mortality rates of 42.7 and 27.6 per 100,000 women respectively [1]. Burundi is not exempted with an annually estimated incidence of 1429 ICC cases and 1080 deaths, corresponding to an annually age-standardised incidence and mortality rates of 49.3 and 39.3 per 100,000 women respectively [1, 6].

Currently, despite the high burden of ICC, there is no national organized cervical cancer screening strategy in Burundi. Pap smear can only be done at the teaching hospital in Bujumbura, Roi Khaled, when gynaecologists propose it to their clients and the latter are able to afford this service. As a result, very few women are screened and few have heard about cervical cancer prevention. The only functional pathology laboratory in the country is hampered by frequent stock out of reagents, very old equipment and insufficient personnel as there are only 2 pathologists and 3 cyto-technicians with only bench training [7].

Therefore, the Burundian health system needs to implement a cervical cancer programme that provides affordable, accessible and acceptable services to ensure adequate coverage to the target population. These services should be provided by skilled and competent health workers. Research and experience have proven that well-organized screening and treatment programmes are highly effective in ICC prevention in LMICs [6, 8, 9]. In order to achieve this, the World Health Organization (WHO) recommends the involvement of existing health personnel *in casu* doctors, nurses, midwives and all available health agents in ICC prevention [10]. To comply with this recommendation, the first step is to make sure that health personnel have appropriate knowledge about cervical cancer control so that they are able to play a sustainable role in ICC control.

The Ministry of Health (MOH) in Burundi, through its non-communicable diseases control programme “PNILMCNT” (Programme National Intégré de Lutte contre les Maladies Chroniques non Transmissibles), has recently developed a cancer national strategic plan 2016–2020, including ICC control. This strategic plan calls for implementation of an ICC screening strategy, typically using Visual Inspection with 5% Acetic acid (VIA)/Visual Inspection with Lugol’s Iodine (VILI) and cytology where feasible, followed by treatment of precancerous lesions with cryotherapy. However, there is a lack of information about the knowledge and practices of the existing health personnel towards ICC prevention. The Burundian health system mostly relies on nurses and General Practitioners (GPs). These GPs are assumed to be the most knowledgeable staff working at District hospitals. They supervise nursing staffs assisting them in all their activities. To our knowledge, there are no available studies assessing the knowledge and practices of GPs about cervical cancer prevention in Burundi.

The aim of this study was to document the knowledge and practices of GPs at district hospital level towards ICC control to inform policy makers and training institutions on their actual knowledge and practices.

## Methods

### Study design

A cross-sectional survey was conducted from February to April, 2015 among all GPs working in the government District hospitals in Burundi.

### Setting, sampling and recruitment

The Burundi health system is composed of 48 government district hospitals. The health staffs at these health districts are composed of GPs and nurses, except in very few hospitals where there are also midwives. During the year 2015, the 48 government district hospitals had a total of 225 GPs. Each district hospital was visited by an investigator during 3 consecutive working days. We aimed to reach all GPs working in district hospitals and all GPs present on site the day of the visit were approached individually and invited to participate in the study. The investigator explained the purpose of the study and after giving their verbal consent, participants were given a questionnaire to fill in. A total of 131 GPs were found at their working place and all accepted to participate in our study. The remaining 94 GPs were on a work assignment elsewhere or on leave and could not be found to be part of the investigation during the timeframe of our survey. Information related to age, sex and professional experience for GPs not present during the 3 days of the visit was also obtained through the management team of the hospital (each personnel has an individual folder kept by the Directorate). Whenever possible, each GP not present were called on his mobile phone to confirm this information.

### Data collection

We found, through literature review, a survey questionnaire and a scoring system assessing knowledge, attitudes and practices of health professionals regarding ICC in Côte d’Ivoire [4]. An expert panel composed out of an epidemiologist, gynaecologist, public health experts and scientists reviewed and adapted some questions from these tools to allow us to capture knowledge and practices of GPs in our context. The adapted questionnaire has been pre-tested on 15 professionals (06 gynaecologists, 04 residents in Gynaecology Obstetrics and 5 GPs) working at the tertiary hospital, Roi Khaled, to check how understandable were the questions and estimate the time required for its full filling in. A Cronbach alpha coefficient, to check for the internal consistency of this tool was 0.63. This tool was found to be satisfactory and was used to collect data.

The questionnaire, consisting of closed (multiple choice) questions, was designed to collect data on demographic characteristics of GPs, their knowledge related to ICC and its prevention options, and their daily practices about proposing cervical cancer screening to their clients.

The knowledge was evaluated using two scores. The first score assessed knowledge of epidemiology and risk factors for cervical cancer. The second score evaluated knowledge of cervical cancer prevention methods, screening and vaccination. The first score was composed of 12 questions and the second score was composed of 17 questions, each worth one point. Regarding GPs practices, participants were asked if they propose screening for cervical cancer to their eligible clients. They were given these choices: never, rarely (less than 20% of their eligible clients), often (20%–60% of the eligible clients) and always. An open question was added to collect reasons for never/rarely proposing a screening test.

After the completion of the questionnaires, data were entered into excel sheets by two different desk clerks. The two databases were compared in order to verify registration errors.

### Statistical analysis

We created two variables: “Knowledge of cervical cancer disease” corresponding to the first score and “Knowledge of cervical cancer prevention” was corresponding to the second one. The first score was composed of 3 questions related to epidemiology and 9 questions on risk factors. The second score was built using 6 questions on preventive methods, 5 questions on screening techniques and 6 questions related to HPV vaccination. Each time a correct answer was given; 1 point was added to the score. For some questions which had two correct answers, if one correct answer was given, 0.5 point was added to the score and if no correct answer was given, the corresponding mark was 0 point (Table 1). The maximum value for the first score was 12 points, equivalent to 100% and 17 points for the second score, also equivalent to 100%. Each score was dichotomized into “Appropriate” when the score was >70% and “Inappropriate” when the score was ≤70%.

**Table 1:** Construction of scores for the assessment of knowledge on cervical cancer among GPs, 2015

Items studied	Topics	Number of questions	Theme of questions	Topic value	Total score	Scale of analysis
Score of knowledge on cervical cancer disease	Epidemiology	3	Incidence	Min = 0 Max = 3	12	0–100%
			Mortality			
			Age			
	Risk factors	9	HPV	Min = 0 Max = 9		
			Sexuality			
			Tabac			
			Multiparity			
Score of knowledge on cervical cancer prevention	Prevention methods	6	Vaccine	Min = 0 Max = 6	17	0–100%
			Screening			
			Treatment of low grade lesions			
	Screening	5	Type of test	Min = 0 Max = 5		
			VIA			
			HPV test			
			Age			
	Vaccination	6	Availability	Min = 0 Max = 6		
			Target			
			Doses			
			names			
			schedule of doses			

Chi^2^ test was used to compare proportions of female participants and non-participants. Fisher’s exact test was used (in case of small numbers) to compare proportions of GPs with appropriate knowledge (both on ICC disease and prevention) among:

1. GPs who never/rarely propose screening test and GPs who often/always propose screening.
2. GPs with professional experience of less than or equal to 1 year and GPs with professional experience of more than 1 year.

Kruskal-Wallis test was used to compare the median age (both years of life and years of professional experience) of participants and non-participants.

A *p*-value <0.05 was considered as statistically significant. The analysis was performed using stata 13 software.

### Ethical aspects

The study protocol was approved by the Burundian National Ethics Committee and we also had an oral agreement of the District Medical Officer (DMO) and/or director of the hospital in each district hospital in which the survey was conducted. Participation in the study was entirely voluntary and participants provided a verbal consent after having been explained the objectives of the study and ensured confidentiality.

## Results

### Demographic characteristics

Out of 48 public hospitals in Burundi, 45 district hospitals were visited; a 3 hospitals had security issues at the time of the survey. A total of 131 GPs (58.2%) of the 225 were present on their duty the day of the visit and all of them (100%) accepted to participate in the study. The median age of the participants was 33 years, (interquartile range [IQR]:32–35). Among the participants, 13 (10%) were female. The median professional experience duration of the participants was 2 years (IQR: 1–3).

### Knowledge of cervical cancer

Overall, 100 participants (76.3%) had appropriate knowledge (score > 70%) on cervical cancer disease. The topics ‘Risk factors’ and ‘Epidemiology’ were best known by the participants – compared with topics related to preventive practices - whereby 68.7% and 64.1% respectively had appropriate knowledge.

More than 90% of the participants knew the 2 most frequent cancers in Burundian women. Similarly, more than 90% knew that early sexual intercourse debut, many sexual partners and Human Papillomavirus (HPV) infection are risk factors for ICC; 89% knew that HPV is isolated in most cervical cancer cases.

On the other hand, only 68.7% of the participants knew the most affected age group; 50.4% knew that high parity is a risk factor for ICC, and only 49.6% knew that smoking is a risk factor for cervical cancer. Only 19 participants (14.5%) knew the 2 most important oncogenic HPVs (Table 2).

**Table 2:** Knowledge of GPs about cervical cancer disease and prevention, Burundi 2015

Items studied	Topics	Question theme	Total score *N* = 131 (%)	Score (>70%) by topic	Score of knowledge (>70%)
Knowledge on cervical cancer disease	Epidemiology	Knew the 2 Most frequent cancer in women ^ǂ^	118(90.1)	84 (64.1%)	100 (76.3%)
		Knew the 2 Most deadly cancer in women^ǂǂ^	99 (75.6)		
		Knew the most affected age group(40-64 years)	90 (68.7)		
	Risk factors	Knew that HPV is a STI	115 (87.8)	90 (68.7%)	
		Knew early sexual debut(<17 years) is a risk factor(RF)	129 (98.5)		
		Knew that many sexual partners is a RF	129 (98.5)		
		Knew that HPV infection is a risk factor	127 (96.5)		
		Knew that multiparity is a risk factor	66 (50.4)		
		Knew that smoking is a risk factor	65 (49.6)		
		Knew that HPV is present in most CC cases	117 (89.3)		
		Knew that HPV can cause condyloma	113 (86.3)		
		Knew the 2 important oncogenic HPV(16–18 types)	19 (14.5)		
Knowledge on cervical cancer prevention	Prevention methods	Knew that cervical cancer is preventable	127 (97.2)	64 (50.4%)	11 (8.4%)
		Aware that vaccine is a preventive method	70 (55.1)*		
		Aware that screening is a preventive method	117 (92.1)*		
		Aware that treatment of CIN is a preventive method	113 (89)*		
		Knew that LEEP is a treatment method for CIN	85 (64.9)		
		Knew cryotherapy is a treatment method for CIN	63 (48.1)		
	Screening	Aware pap smear is a screening method	126 (96.2)	18 (13.7%)	
		Aware VIA is a screening method	20 (15.3)		
		Aware VILI is a screening method	41 (31.3)		
		Aware HPV test is a screening method	108 (82.4)		
		Knew age to start cervical cancer screening	20 (15.3)		
	Vaccination	Aware of existence of HPV vaccine	86 (65.6)	1 (0.8%)	
		Knew target group for HPV vaccine	90 (68.7)		
		Knew target group chosen by Burundi	14 (10.7)		
		Knew names of commercially available vaccines	6 (4.6)		
		Knew required doses to complete HPV vaccination	4 (3.1)		
		Knew timeline of HPV vaccination	1 (0.8)		

*N = 127, ^ǂ:^ Cervical cancer and breast cancer; ^ǂǂ:^ Cervical cancer and breast cancer

### Knowledge of cervical cancer prevention

Overall, only 11 participants (8.7% of respondents) had appropriate knowledge (score > 70%) on cervical cancer prevention. The items related to “Vaccination” and “screening” were less known by our participants whereby only 1 participant (0.8%) and 18 participants (13.7%) respectively had appropriate knowledge. Regarding prevention in general, 55% of the participants was aware that HPV vaccination is a preventive method of ICC and only 48.1% knew that cryotherapy is a treatment method for cervical intraepithelial neoplasia. In terms of screening, only 15.3% was aware that Visual Inspection with Acetic acid (VIA) is a screening method, compared with 82% knowing that HPV screening tests exist. With regard to vaccination, only 10.7% knew the target age group chosen by the Burundi ministry of Health for HPV vaccine (i.e. 9-13 years); 4.6% knew the names of commercially available vaccines; 3.1% knew the required doses for HPV vaccination completion and only one participant knew the timeline of HPV vaccination (Table 2).

### Practice of GPs

Our results showed that 87% of the participants never or rarely (42.7% and 44.3% respectively) propose screening to their clients. None of the participants reported to always propose screening test to his/her clients. Only 13% reported to often propose the screening test to their clients (Table 3).

**Table 3:** Practices of GPs and reasons for never/rarely proposing screening, Burundi 2015

Variable	*n* (%)
Propose screening test (*N* = 131)	
Never	56 (42.7)
Rarely	58 (44.3)
Often	17 (13)
Always	0 (0)
Have heard about VIA/VILI (*N* = 131)	48 (36.6)
Have already performed VIA/VILI (*N* = 131)	2 (1.5)
Have heard about HPV screening (*N* = 131)	90 (68.7)
Reasons for never or rarely proposing screening (*N* = 114)	
Screening center not available	86 (75.4)
Ignorance of GPs	56 (49.1)
Financial obstacles	32 (28.1)
women don’t come	16 (14)
GPs overloaded	8 (7)
Late response of the pathology laboratory	1 (0.9)
Thoughts of GPs about ICC prevention (*N* = 131)*	
Think radiotherapy is a treatment of CIN	52 (39.7)
Think CIN can be treated with hysterectomy	35 (26.7)
Think vaginal echography is a screening method ICC	25 (19.1)
Think RX pelvis is a screening method for cervical cancer	4 (3.1)

*The respondents who gave these answers are wrong

Only 36.6% of the participants have heard about VIA/VILI and among them, only 2 (1.5%) have already performed VIA/VILI.

The most reported reasons for never or rarely proposing screening test (Table 2, *N* = 114)) were:

1. Screening centre not available at (or around) their working places (75.4% of respondents),
2. Ignorance of GPs (49.1% of respondents) (meaning GPs not trained on cervical cancer screening techniques, GPs forget to ask screening because they don’t have the habit of doing so, GPs think that the prevalence of CC is very low and thus not worth to worry about it, GPs are not sensitised about cervical cancer, GPs think that the management of CIN is not feasible in Burundi);
3. Financial barriers for their clients (28.1%);
4. Women don’t come for screening (14%)(they come at a late stage, they hide their early sexual debut, women not sensitised on the interest of doing CC screening)
5. GPs are overloaded (7%)
6. and the late response of the pathology lab (0.9%).

Among our participants, we noticed high proportions of wrong answers: 39.7% of the participants thought that CIN could be treated with radiotherapy and 26.7% suggested treating CIN with hysterectomy; 19.1% thought that vaginal ultrasound is a screening method and 3.1% thought that X-ray of the pelvis is a screening method for cervical cancer.

We sought to know if there would be an association between knowledge of ICC (disease or prevention) and practice (=proposing screening for cervical cancer), and found no association (*p* = 0.18; *p* = 0.14 respectively) suggesting that those who have appropriate knowledge (score > 70%) about ICC disease or prevention are not more proposing screening to their clients than those who have less knowledge.

However, we found an association between professional experience and knowledge of cervical cancer disease. Those having less experience have better knowledge on cervical cancer disease than those with more experience (>1 year) (*p* = 0.038). We did not observe any significant association between experience and knowledge of ICC prevention (Table 4).

**Table 4:** Association between knowledge and practice and professional experience

Variable	Knowledge disease (*n* = 131)			Knowledge prevention (*n* = 127)		
	Score > 70%	Score ≤ 70%	*p*-value	Score > 70%	Score ≤ 70%	*p*-value
Propose screening						
Never/rarely	85 (74.6)	29 (25.4)	0.18*	8 (7.2)	103 (92.8)	0.14*
Often/always	15 (88.2)	2 (11.8)		3 (18.8)	13 (81.2)	
Experience						
≤ 1 year	42 (85.7)	7 (14.3)	0.038*	4 (8.7)	42 (91.3)	0.61*
> 1 year	58 (70.7)	24 (29.3)		7 (8.7)	74 (91.4)	

*p Fisher exact test

## Discussion

This study is one of the few presenting the knowledge and practices of general practitioners towards cervical cancer disease and its prevention and the first one in Burundi. These physicians working at district hospital level are involved in women’s primary care and are well trusted by their clients. We undertook this study in order to inform policy makers and training institutions about their level of knowledge and practices on cervical cancer disease and prevention. More than half of our participants had good knowledge about risk factors and epidemiology of cervical cancer. On the other hand, knowledge about screening techniques and vaccination was very low (only 13.7% and 0.8% respectively had appropriate knowledge i.e. a score > 70%). Our study revealed important gaps in the cervical cancer prevention knowledge whereby only 8% of the participants had appropriate knowledge about prevention (score > 70%). Our findings are in line with results from others studies on health professionals pointing out inadequate knowledge about cervical cancer prevention [11, 12]. This low knowledge on CC prevention is probably due to the fact that invasive cervical cancer has been neglected: there is no national programme for screening or vaccination and GPs are not sensitized on ICC prevention activities. Much emphasis has been put on other diseases like HIV/AIDS, tuberculosis and malaria for which different control programmes exist and are funded by government and its partners; this is not the case for cervical cancer. Low knowledge in cervical cancer prevention has also been reported in studies done in other settings [13–15]. In Tunisia, in a study done among medical students at the end of their curriculum, pointed out that more than 70% of the participants did not know when to start cervical cancer screening and neither did they know the periodicity. The study concluded that cervical cancer control training was insufficient and should be improved and restructured [16].

Indeed, the low knowledge level in our study points out insufficient training at the faculties of Medicine in Burundi. In fact, training programmes in cancer control in general and cervical cancer in particular, are poorly or not at all developed. Cervical cancer control is briefly outlined in the module of Gynaecology. The practical training in cervical cancer control (screening and vaccination) is not available in Burundi. Roi Khaled, the main University teaching hospital in Burundi, does not have any cervical cancer control unit. It is neither equipped with a cryotherapy machine, LEEP machine, colposcopy machine nor practices HPV vaccination. Moreover, the health providers stationed in this department are not trained in screening for cervical cancer, HPV vaccination and colposcopy.

To our knowledge, the same situation prevails in all public and private hospitals, including at tertiary level.

Our study showed a significant association between knowledge of cervical cancer disease and professional experience whereby the proportion of GPs with less experience having appropriate knowledge on ICC disease (score > 70%) was significantly higher than the more experienced GPs. This would suggest that GPs with more professional experience know less than the younger GPs. We hypothesised that this could be explained by the fact that the questions about epidemiology and risk factors are theoretical knowledge and the younger GPs are more likely to know it because they recently graduated from faculties. Moreover, there is no organised in-service training for GPs on this topic. Importantly, the study revealed no association between appropriate knowledge of cervical cancer (disease and prevention) and practice (=proposing screening). This result suggests that GPs with appropriate knowledge about cervical cancer do not propose more screening tests to their clients than those with insufficient knowledge. The reasons for that situation are likely the absence of screening centre at/or around their working places and other reasons as it has been reported (Table 3).

The present study provides a preliminary identification of gaps in physician’s knowledge about cervical cancer prevention and its epidemiology. Our ultimate hope is that it will allow tailoring a more targeted educational programme. The key areas of focus should include the HPV vaccination, cervical cancer screening techniques and prevention methods.

The study shows that majority of the participants never or rarely propose to their clients a screening test for cervical cancer and as expected, the reported reasons for that are screening centre not available at/or near their working places or even for some in the whole country (75%); 50% of GPs acknowledged their ignorance for cervical cancer screening.

For GPs who dare to ask for a pap smear, they are disappointed by the late response from the pathology laboratory. In fact, this is also another big challenge in our context where the pathology lab suffers from many malfunctioning (insufficient qualified staff, frequent stock outs of reagents, inadequate equipment, etc.). This is in line with results from other low resource settings pointing out the ineffectiveness of cytology-based screening programs in resource constraints settings [11, 14]. This situation has led to the development of other alternative screening modalities for LMICs such as VIA and rapid, low-cost HPV DNA testing that do not require follow-up visits and have been successfully used in low and middle resource settings [17, 18]. However, our results revealed that the vast majority of GPs (64%) had never heard of the VIA/VILI screening methods; and importantly, only 2 participants had already performed VIA/VILI. This situation calls for attention because these screening modalities are likely the most feasible in our context. Therefore, training and education about these alternative approaches is critical and should be made a training priority for health care providers involved in women’s primary care as some steps are being taken to scale up VIA cervical cancer screening in Burundi.

Surprisingly, the study revealed a high proportion of wrong answers of our participants about cervical intraepithelial neoplasia treatment and screening techniques (Table 3). In line with what have been described above, this also denotes a lack of training.

### Study limitations

The participation rate was only 58.2% of the total GPs working at district level (three district hospitals were not visited due to security issues and some general practitioners at district hospitals visited were not at their working place). We think this limitation does not have important implications as the participants and non participants to our study had roughly the same characteristics (Table 5).

**Table 5:** Characteristics of participants and non participants

Characteristic	Participants (*N* = 131)	Non-participants (*N* = 94)	*P*-value
Age, years			
Median (IQR)	33 (32–35)	34 (32–37)	0.04*
Sex			
Female (%)	13 (10%)	15 (16%)	0.18**
Professional experience, years			
Median (IQR)	2 (1–3)	2.5 (1–5)	0.19*

*=Kruskal-Wallis test;

**= Chi^2^test

A second limitation could be related to the cronbach alpha coefficient of our tool of 0.63 which might be considered as low by some authors. However, other authors support the hypothesis that Cronbach’s alpha values higher than 0.60 could be acceptable [19]**.**

## Conclusion

A very low knowledge level about cervical cancer prevention, screening and treatment exists among Burundian general practitioners. GPs practices are also suboptimal. A high proportion of wrong thoughts related to CC screening and treatments have also been documented. If knowledge among GPs is so low, knowledge among nurses should also be investigated since they can greatly assist in cervical cancer control. We therefore recommend pre- and in-service training of GPs, among others, before setting up any public health intervention on cervical cancer control. If this recommendation is met and health services strengthened with investment in adequate infrastructure, cervical cancer control programme can then be successfully implemented.

## Abbreviations

CC: Cervical Cancer
CIN: Cervical Intraepithelial Neoplasia
DNA: Desoxyribonucleic Acid
GPs: General Practitioners
HIV: Human Immunodeficiency Virus
HPV: Human Papillomavirus
ICC: Invasive Cervical cancer
IQR: Interquartile Range
LMICs: Low and Middle Income Countries
PNILMCNT: Programme National Intégré de lutte contre les Maladies Chroniques non transmissibles
RF: Risk Factor
STI: Sexually Transmitted Infection
VIA: Visual Inspection with 5% Acetic acid
VILI: Visual Inspection with Lugol’s Iodine
WHO: World Health Organization
